# Decreasing prevalence of Hepatitis B and absence of Hepatitis C Virus infection in the Warao indigenous population of Venezuela

**DOI:** 10.1371/journal.pone.0197662

**Published:** 2018-05-25

**Authors:** Ruth Y. Blanco, Carmen L. Loureiro, Julian A. Villalba, Yoneira F. Sulbarán, Mailis Maes, Jacobus H. de Waard, Héctor R. Rangel, Rossana C. Jaspe, Flor H. Pujol

**Affiliations:** 1 Laboratorio de Virología Molecular, Centro de Microbiología y Biología Celular, Instituto Venezolano de Investigaciones Científicas, Caracas, Venezuela; 2 Laboratorio de Tuberculosis, Instituto de Biomedicina, Universidad Central de Venezuela, Caracas, Venezuela; Centre de Recherche en Cancerologie de Lyon, FRANCE

## Abstract

Prevalence and molecular epidemiology studies for hepatitis B (HBV) and C (HCV) virus are scarce in Warao Amerindians from Venezuela, where an epidemic of human immunodeficiency virus type 1 (HIV-1) has recently been documented. To carry out a molecular epidemiology analysis of hepatitis B (HBV) and C (HCV) virus in Warao individuals from the Delta Amacuro State of Venezuela. A total of 548 sera were tested for serological and molecular markers for HBV and HCV. The prevalence of active infection (presence of HBV surface antigen, HBsAg), exposure to HBV (presence of Antibody to HBV core antigen, anti-HBc) and anti-HCV, was 1.8%, 13% and 0% respectively. HBV exposure was significantly lower in men below 18 years old and also lower than rates previously reported in other Amerindian communities from Venezuela. Thirty one percent (31%, 25/80) of individuals without evidence of HBV infection exhibited anti-HBs titer ≥ 10U.I / ml, being significantly more frequent in individuals younger than 20 years. A higher HBV exposure was observed among HIV-1 positive individuals (33% vs 11%, p <0.005). A high prevalence of occult HBV infection was also observed (5.6%, 11/195). Phylogenetic analysis of S gene and complete HBV genomes showed that F3 is the only circulating subgenotype, different from the F2 subgenotype found in 1991 in this population. These results suggest a recent introduction of subgenotype F3, with a low divergence among the isolates. These results highlight the importance of molecular epidemiology studies for viral control, and support the effectiveness of vaccination in reducing transmission of HBV.

## Introduction

Infections caused by Hepatitis B (HBV) and C virus (HCV) are a major public health problem around the world [1]. In Venezuela, HBV active infection prevalence is relatively low (< 2%) [2], except in Amerindian populations [3]. Previous studies in Venezuelan indigenous communities have shown high rates of prevalence of active HBV infection from 0–65% and HBV exposure from 15.5–80.5%, with a great variation between different indigenous communities [4, 5, 6, 7]. In addition to the classical HBV infection, HBV occult infection (OBI) appears to be frequent in Amerindians [7, 8]. OBI is characterized by the long-lasting persistence of HBV DNA in the liver, with detectable or undetectable HBV in the serum, in the absence of HBV surface antigen (HBsAg) [9]. OBI can lead to an increase in severe chronic manifestations and accelerate liver injury in patients with chronic liver disease including chronic HCV infection [10, 11, 12].

Up to 10 HBV genotypes (A-J) and multiple subgenotypes have been described [13]. Genotype F is the most divergent of the HBV genotypes, autochthonous to South America and highly predominant in Venezuela, particularly in Amerindians [14, 15, 16]. Four subgenotypes, F1 to F4, have been described inside HBV genotype F [17]. A new subgenotype F5 has been proposed in Panamanian blood donors [18]. In Venezuela, F3 is the most common subgenotype followed by subgenotype F2; it is uncommon to find more than one subgenotype circulating in a specific Amerindian community [14, 15].

It is estimated that 1.5% of the general population has antibodies against the virus (anti-HCV), while in Amerindian population a seroprevalence up to 2.1% has been reported. However, in many of these cases, the reactivity of antibodies has not been associated with the presence of viral RNA [19]. While HBV is endemic in Amerindian communities, HCV is more an imported virus in them, with low or null prevalence [19, 20, 21, 22].

Most epidemiological studies of HBV and HCV in Venezuelan Amerindians have been oriented to Yanomami and Piaroa population [4, 5, 6, 7, 19, 20]. Although HBV infection is highly frequent among Amerindians, the prevalence and rate of acquisition of this infection varies between each community [5, 7]. Little information is available about HBV infection and on the eventual presence of HCV in Warao population, where an epidemic of Human immunodeficiency virus type 1 (HIV-1) infection has already been documented [23, 24].

Warao Amerindians inhabit the Orinoco River Delta in Northeastern Venezuela and adjacent areas. It is the second most numerous indigenous tribe, with a population of 48.771 individuals [25]. Over time, different factors have forced this population to migrate to the nearest urban centers in search of improvements, which consequently has allowed the introduction of diseases to which they had never been exposed [26, 27, 28]. The aim of this study was evaluate the prevalence and genetic diversity of the HBV and HCV in Warao Amerindians Venezuela.

## Materials and methods

### Blood samples

This study was approved by The Bioethical Committee of Instituto Venezolano de Investigaciones Cientificas (IVIC), Servicio de Atención y Orientación al Indígena (SAOI) (Office in charge of Indigenous Health in Delta Amacuro State) and the Local Office of Health Programs of Delta Amacuro State. Consent of villages was obtained through meetings with Warao community councils and written informed consent of each individual was verbally translated into the Warao language. A total of 548 blood samples from Warao individuals (287 male and 261 female, median age 27 years) living in ten villages of Delta Amacuro state from Venezuela (CañoYeri n = 2, Iburuina n = 6, Ibute-Guayo n = 18, Isla de Jobure n = 24, Jeukubaka n = 27, Jobotoboto n = 23, Jobure de Guayo n = 84, Jokabanoko n = 2, San Francisco de Guayo n = 328 and Usidu n = 34), were obtained during year 2011 and stored at −20°C until use. These samples were previously tested for HIV-1 positivity, finding a high prevalence (9.55%) of infection [23].

### HBV and HCV serologic markers

Serum samples were tested for Hepatitis B surface antigen (HBsAg), total antibodies against HBV core antigen (anti-HBc) and antibodies against HCV (anti-HCV), using immunoenzymatic assays (ELISA) (Biokit S.A Barcelona, Spain). HBV and HCV samples exhibiting an optical density above or near the tests cut-off value, were then tested using a third generation immunoenzymatic assays (ELISA) according to the manufacturers instructions (DiaSorinS.p.A UK Branch and Bio-Rad France) and by polymerase chain reaction (PCR). Eighty serum samples selected at random, without serological and molecular evidence of infection by HBV, were tested from antibodies against HBsAg (anti-HBs) using ELISA ETI-AB- AUK-3 (DiaSorinS.p.A. Italy).

### Polymerase chain reaction (PCR) and sequencing

A total of 188 serum samples (10 positive HBsAg, 68 negative HBsAg / positive anti-HBc and 110 samples HBsAg and anti-HBc negative randomly selected) were tested for the presence of HBV-DNA as previously described [15, 29]. Sequence of partial S gene or complete genome was performed as previously described [30, 31] and were deposited in GenBank under the accession numbers MH051986- MH051998.

HCV RNA was detected by amplification of the 5´ non-coding region (5´NC) using reverse transcription and nested polymerase chain reaction (RT-PCR) [32].

### Phylogenetic and sequence analysis

Sequence alignment and phylogenetic analysis were conducted using the Molecular Evolutionary Genetics Analysis version 7 software (MEGA 7.0.26) [33]. The phylogenetic trees were performed using the Neighbor Joining method (1000 bootstrap replicas) with genetic distances evaluated with Kimura 2 parameters corrections. Reference sequences from the different genotypes and subgenotypes were included in the phylogenetic analysis, as well as sequences closely related to the Warao sequences analyzed, obtained by BLAST evaluation. Electropherotypes were also visually inspected to detect the presence of variants in specific nucleotides, associated with stop codons or other relevant mutations or polymorphisms.

The presence of mutations in the ‘‘a” antigenic loop and the major hydrophilic region (MHR) were analyzed from amino acids 100 to 181 of HBsAg.

### Statistical analysis

Statistical differences were evaluated by the Chi-Squares test with Yates correction, or Fisher Exact test (when a number under 5). P values less than 0.05 were considered significant. All analysis were done using Epi Info program, version 3.5.3 (Centers for Disease Control and Prevention, Atlanta, GA).

## Results

### Prevalence of HBV and HCV infection in Amerindian Warao population of Venezuela

A total of 548 serum samples of individual from ten Warao communities were tested for HBV and HCV serological markers. The overall prevalence of HBsAg, anti-HBc and HCV infection (anti-HCV and HCV RNA positivity) were 1.8% (10/548), 13% (71/548) (Table 1) and 0% (0/548) respectively. The prevalence of HBsAg was similar in both genders (2%, 6/287 in males vs. 1.5%, 4/261 in females), without any significant differences in age groups of this population. The prevalence of total anti-HBc was significantly higher (p<0.0001) in males (18%, 52/287) compared with females (2%, 4/261), with a higher prevalence in men over 18 years old (p = 0.03) (Table 1).

**Table 1:** Serological prevalence of HBV markers (HBsAg and anti- HBc) by age group in Amerindian Warao communities from Delta Amacuro State, Venezuela.

	Positive HBV serological markers			
	HBsAg (%)		Anti-HBc (%)	
Age (years)	F	M	F	M
<18	2/68 (3)	1/76 (1)	2/68 (3)	7/76 (9)
19–30	2/90 (2)	2/86 (2)	8/90 (9)	15/86 (17)
31–50	0/82 (0)	2/92 (2)	7/82 (9)	21/92 (23)
≥ 51	0/21 (0)	1/33 (3)	2/21 (10)	9/33 (27)
Total	4/261 (2)	6/287 (2)	19/261 (7)	52/287 (18)
**Total F+M**	**10/548 (1.8)**		**71/548 (13)**	

Numbers into parentheses denote the prevalence percentage. F: female, M: male.

### Immunity acquired by vaccination

**Fig 1:**
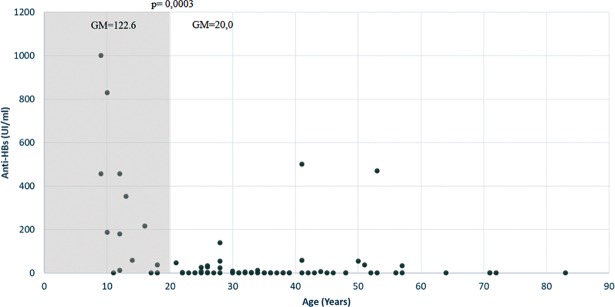
Distribution anti-HBs titres (IU/ml) by age in population Amerindian Warao. The gray shadow indicates the group under 20 years of age with anti-HBs titers significantly higher (p = 0.01) than those obtained in the population over 20 years of age. GM refers to the geometric mean of the anti-HBs titer.

In order, to evaluate the immune status of Warao population, eighty serum samples selected at random, without serological and molecular evidence of infection by HBV were tested from antibodies against HBsAg (anti-HBs). Thirty one percent (31%, 25/80) of individuals exhibited anti-HBs titer ≥ 10U.I / ml, without significant difference between female and male (women 39%, 17/44 vs. men 22%, 8/36). A higher proportion and titer of anti-HBs was found in the age group equal to or lower than 20 years, with respect to the older age group (p = 0.0003 and p = 0.01 respectively) (Fig 1).

### Co-infection with HIV

From the previous data on HIV-1 infections in this same study population [23], a higher prevalence of HBV exposure with respect to HIV-1 was observed (13%, 71/548 anti-HBc positive vs. 7.1%, 39/548 HIV-1 positive, (p = 0.002). From the 71 individuals exposed to HBV, 13 (18%) were infected with HIV-1. The co-infected population was composed entirely of male individuals, representing 25% (13/52) of the male population exposed to HBV, compared to female population where no co-infection was detected. The prevalence of HBV exposure was significantly higher (p = 0.0002) in HIV-infected individuals (33%, 13/39) than in HIV-negative ones (11%, 58/509).

### Detection of HBV occult infection

**Fig 2:**
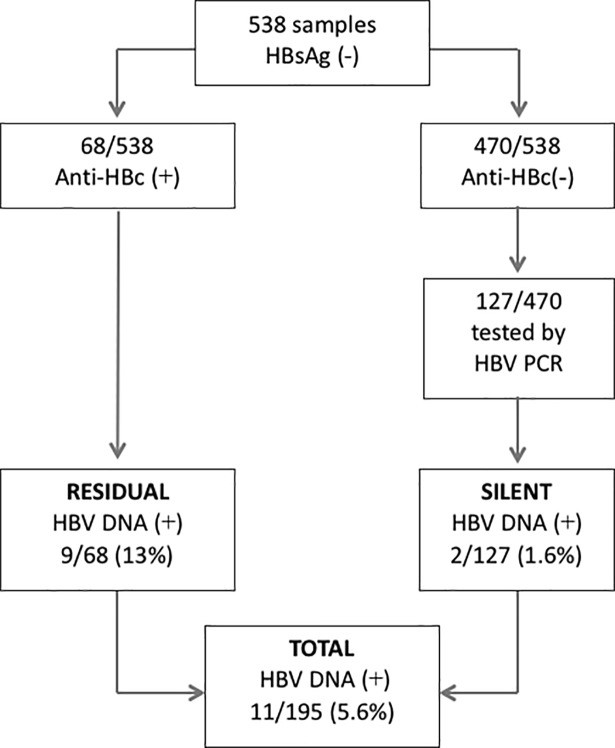
Occult hepatitis B Infection (OBI) prevalence in Warao Amerindians. Positive and negative samples are represented with + o–symbol respectively.

A total of 205 sera (10 HBsAg positive and 195 HBsAg negative) from Amerindian Warao population were analyzed for the presence of HBV DNA. The S gene of HBV DNA was amplified in all HBsAg positive samples (10/10), one of them also HIV-1 positive. OBI was 5.6% (11/195) of a subgroup (195/538) of the Warao population, with 13% (9/68) of denominated residual OBI (with anti-HBc positivity) and 1.6% (2/127) of silent one (without either HBsAg or anti-HBc positivity) (Fig 2). No significant difference was observed in the OBI prevalence according to sex: HBV DNA was detected in the sera of 4/85 females and 7/110 males. Of the population evaluated for OBI, 19.5% (38/195) individuals were infected with HIV-1 and only one was positive for HBV DNA (residual OBI). The difference in prevalence of OBI between the HIV- (6.4%, 10/157) and HIV+ (2.6%, 1/38) population was not significant. In this sense, the active HBV/HIV co-infection prevalence was 5.1% (2/39).

### HBV genetic characterization

**Fig 3:**
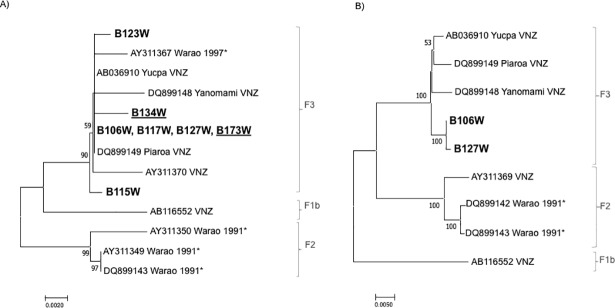
Phylogenetic tree of HBV isolated from Amerindian Warao population. A) Phylogenetic analysis of HBV S region (658 bp) and B) of two complete genome HBV isolated of Amerindian Warao population. Genetic distance was estimated by Kimura two parameters correction, MEGA version 7.0.26. Phylogenetic tree was constructed with the Neighbour-joining method. Numbers at each node correspond to bootstrap values (>50%) obtained with 1000 replicates. The prototype strains are designated by their GenBank accession number, and country or ethnic group, except the Warao isolates of this study (bold). VNZ: Venezuela. *These Warao isolates were obtained between the years 1991–1997 and analyzed in a previous study [34]. The sequences of occult HBV infection are underlined. Six isolates for which only a 330 pb fragment could be amplified are not included in the tree.

To determine the genetic diversity of HBV among Warao Amerindians 13 partial sequences of the S gen and two complete genomes were analyzed. Phylogenetic analysis of the partial HBV S region (from 9 active infections and 4 occult infections) and two complete genomes showed that all the strains belonged to HBV subgenotype F3 (Fig 3A). The 13 strains studied were closely related to each other, with 99.5–100% identity. The isolates of this study were closely related to a F3 HBV isolate found during year 1997 among Warao population [34]. Complete genome analysis of two Warao isolates confirmed the close identity (99.9%) found in this group of sequences (Fig 3B).

Substitutions related to HBV occult phenotypes [35], with vaccine escape mutants impaired virion and/or S protein secretion in vitro [36, 37, 38] or YMDD variants in polymerase [39, 40], were not detected (data not shown).

## Discussion

In the Warao Amerindians population, prevalence and molecular epidemiology studies are scarce for HBV and HCV. In this study, HBV active infection and exposure (1.8% and 13%) were lower than the ones reported in previous studies in 1973 (5% and 34%) and 1991 (8.37% and 30.9%) [41, 42]. No serological or molecular evidence of HCV infection was observed, in contrast to unique previous study that reported 2.5% of exposure to HCV in 775 samples from Warao individuals, without mentioning the study community [19]. HBV and HCV prevalence might be influenced by the migration process of the Warao population in the mid-1990s towards urban populations [26, 27]. In any case, the possible introduction of HCV does not seem to have spread in the Warao population, unlike that was observed for example for HIV-1 [23].

The higher HBV exposure prevalence in the Warao population over 18 years might suggest that sexual transmission is the main route of HBV infection in this population. The higher exposure to HBV found in men might be attributed to practice of sex between men [43]. In general, both active infection and exposure to HBV found in Warao population were significantly lower (p <0.00001) than those reported in other Amerindian population in Venezuela, both in Yanomami (14.3% and 58%, respectively) and Piaroa (5.1% and 27.4%, respectively) [5]. In particular, the acquisition of anti-HBc was significantly lower in Warao respect to Yanomami, independently of the age group. These differences may be related to diverse sociocultural practices between these communities. In Yanomami, the initiation of sexual relations at an early age [44] and rituals that involve bleeding and scarification are the main risk factors associated to HBV infection [45, 46]. In the Piaroa population the dental extraction and surgeries are common [6], while Warao do not have rituals that involve bleeding or scarification [47]. In addition, vaccination may play a significant role in reducing the exposure to HBV in the Warao, since the low prevalence of infection HBV in young individuals was accompanied by a higher prevalence and titers of antibodies to anti-HBs. The major proportion of antibodies in the individuals under 20 years suggests a lower prevalence of infection over the course of the next years; however, there is a population currently susceptible to HBV infection that needs to be vaccinated. Although in vaccinated individuals it is possible to detect occult HBV infection (OBI) or escape mutants to the vaccine [48], the beneficial effect of vaccination in reducing the burden of infection by this virus is well documented [49, 2].

As was previously mentioned, the health of Warao population is widely affected by HBV, as well as by HIV-1 [23], which shares the same transmission routes. The HBV exposure prevalence in HIV-1 positive patients found in this study (33%), particularly in the male population, was higher than the one reported in the urban population of Venezuela (14%) and Colombia (12%) [50, 51].

OBI is frequent in Amerindian populations of Latin America, probably due to the immune compromise caused by multiple bacterial infections such as tuberculosis, parasitic infections, malnutrition and HIV-1 co-infection, exhibited by these populations [52, 53, 7]. The immune compromise in different contexts plays an important role in the expression of escape variants of HBV [10, 54]. The overall OBI prevalence in Warao population is similar to that reported in others Amerindian population of countries such as Argentina (6.5%) [55], but lower that the one found in Amerindian communities from Colombia (23.5 to 25%) [56]. The residual and silent prevalence of OBI in Warao population, were significantly lower (p = 0.003 and p < 0.0001, respectively) than the one reported in the Piaroa population (52% residual and 23% silent OBI) [8], that is in concordance with the higher HBV infection prevalence present in the Piaroa population. However, the residual OBI prevalence (13%) was higher than that reported to urban blood donor population of Venezuela (6%) [48]. In HIV-1 positive Warao patients, the residual OBI prevalence was similar to that reported in chronic HIV-1 patients living in urban areas from Venezuela (7%) [57] or Colombian HIV-1 patients (8.7%) [51] but lower than that described in naïve HIV-positive patients living in urban areas from Venezuela (18%) [50].

Phylogenetic analysis showed the presence of F3 as the unique circulating HBV subgenotype in the Warao population, closely related to HBV isolates circulating in other Amerindian population from Venezuela, Piaroa and Yanomami [8]. In a previous study, four HBV isolates were identified, three subgenotype F2 from 1991 and one F3 from 1997 [34]. These data suggest a possible displacement of the F2 subgenotype detected in the early 90s by the F3 subgenotype detected in 1997 and in 2011 years (sampling date of this study). Warao population might be have been exposed to subgenotype F3 HBV during the migration process experienced in the early nineties, as mentioned previously [26, 27]. The eventual displacement of subgenotype F2 by F3 in this population might be favorable, since HBV F3 infection seems to be associated with a lower severe liver disease compared to subgenotype F2 [58]. HBV subgenotype F3 might have been introduced in the Warao population before HIV-1, due to that HBV-F3 was present at least since 1997 [34], while the probable date of introduction of HIV-1 was around 2002 [23].

This study reflects the importance of the epidemiological and molecular analysis in Amerindian populations in order to establish effective strategies for the control and eradication of HBV, and highlights the positive effect of vaccination against HBV in high-risk populations at all ages to achieve greater immunization and decreasing transmission.

## References

[1] World Health Organization. Global hepatitis report, 2017. 2017. 62 p. Available from: http://apps.who.int/iris/bitstream/10665/255016/1/9789241565455-eng.pdf?ua=1.

[2] Zampino R, Boemio A, Sagnelli C, Alessio L, Adinolfi LE, Sagnelli E, et al Hepatitis B virus burden in developing countries. World J Gastroenterol. 2015;21(42):11941–53. doi: 10.3748/wjg.v21.i42.11941 26576083 10.3748/wjg.v21.i42.11941 PMC4641116

[3] Pujol FH. Biología de los Virus de Hepatitis. Acta cient. Soc. Venez. Bioanalistas Esp; 6(1/2): 5–12, 2000.

[4] Monsalve-Castillo F, Echevarría JM, Atencio R, Suárez A, Estévez J, Costa-León L, et al Alta prevalencia de la infección por el virus de hepatitis B en la comunidad indígena Japreira, Estado Zulia, Venezuela High prevalence of hepatitis B infection in Amerindians in Japreira, Zulia State, Venezuela. Cad. Saúde Pública. 2008; 24(5): 1183–1186. 18461250 10.1590/s0102-311x2008000500028

[5] Duarte MC, Cardona N, Poblete F, González K, García M, Pacheco M, et al A comparative epidemiological study of hepatitis B and hepatitis D virus infections in Yanomami and Piaroa Amerindians of Amazonas State, Venezuela. Trop Med Int Health. 2010;15(8):924–933. doi: 10.1111/j.1365-3156.2010.02560.x 20561309 10.1111/j.1365-3156.2010.02560.x

[6] Cardona NE, Duarte MC, Poblete F, Figueroa G, del Valle K, García DM, et al Prevalencia y riesgo de infección por Hepatitis B en población indígena de la Cuenca del Cataniapo. Estado Amazonas, Venezuela. Bol. Venez. infectol. 2015; 26(2): 131–136.

[7] Pujol FH, Cardona N, Loureiro CL, Jaspe R, Chemin I. Hepatitis B Occult Infection in Indigenous Populations from Latin America. JSM Hepat. 2016; 1(1): 1004–1012.

[8] Cardona NE, Loureiro CL, Garzaro DJ, Duarte MC, García DM, Pacheco MC, et al Unusual presentation of hepatitis B serological markers in an Amerindian community of Venezuela with a majority of occult cases. Virol J. 2011;8:527 doi: 10.1186/1743-422X-8-527 22152023 10.1186/1743-422X-8-527 PMC3253065

[9] Hollinger FB, Sood G. Occult hepatitis B virus infection: a covert operation. J Viral Hepat. 2010;17(1):1–15. doi: 10.1111/j.1365-2893.2009.01245.x 20002296 10.1111/j.1365-2893.2009.01245.x

[10] Chemin I, Trépo C. Clinical impact of occult HBV infections. J Clin Virol. 2005 12; 34 Suppl 1:S15–21. . 16461218 10.1016/s1386-6532(05)80005-8

[11] Squadrito G1, Cacciola I, Alibrandi A, Pollicino T, Raimondo G. Impact of occult hepatitis B virus infection on the outcome of chronic hepatitis C. J Hepatol. 2013;59(4):696–700. doi: 10.1016/j.jhep.2013.05.043 23751755 10.1016/j.jhep.2013.05.043

[12] Covolo L, Pollicino T, Raimondo G, Donato F. Occult hepatitis B virus and the risk for chronic liver disease: a meta-analysis. Dig Liver Dis. 2013;45(3):238–244. doi: 10.1016/j.dld.2012.09.021 23146778 10.1016/j.dld.2012.09.021

[13] Araujo NM, Waizbort R, Kay A. Hepatitis B virus infection from an evolutionary point of view: how viral, host, and environmental factors shape genotypes and subgenotypes. Infect Genet Evol. 2011;11(6):1199–1207. doi: 10.1016/j.meegid.2011.04.017 21539937 10.1016/j.meegid.2011.04.017

[14] Devesa M, Pujol FH. Hepatitis B virus genetic diversity in Latin America. Virus Res. 2007;127: 177–184. doi: 10.1016/j.virusres.2007.01.004 17280734 10.1016/j.virusres.2007.01.004

[15] Devesa M, Loureiro CL, Rivas Y, Monsalve F, Cardona N, Duarte MC, et al Subgenotype Diversity of Hepatitis B Virus American Genotype F in Amerindians From Venezuela and the General Population of Colombia. J Med Virol 2008; 80: 20–26. doi: 10.1002/jmv.21024 18041024 10.1002/jmv.21024

[16] Nakano T, Lu L, Hu X, Mizokami M, Orito E, Shapiro C, et al Characterization of hepatitis B virus genotypes among Yucpa Indians in Venezuela. J Gen Virol. 2001;82(Pt 2):359–365. doi: 10.1099/0022-1317-82-2-359 11161274 10.1099/0022-1317-82-2-359

[17] Kramvis A. Genotypes and genetic variability of hepatitis B virus. Intervirology. 2014;57(3–4):141–150. doi: 10.1159/000360947 25034481 10.1159/000360947

[18] Martínez AA, Zaldivar YY, CSS-NAT Group, De Castillo Z, Ortiz AY, Mendoza Y, et al High diversity of hepatitis B virus genotypes in Panamanian blood donors: a molecular analysis of new variants. PLoS One. 2014;9(8):e103545 doi: 10.1371/journal.pone.0103545 25093674 10.1371/journal.pone.0103545 PMC4122375

[19] Aguilar MS, Cosson C, Loureiro CL, Devesa M, Martínez J, Villegas L, et al Prevalence of infection with hepatitis C virus in Venezuela, as assessed with an immuno-assay based on synthetic peptides. Ann Trop Med Parasitol. 2001 3;95(2):187–195. doi: 1080/00034980120042944 11299125

[20] Blitz-Dorfman L, Monsalve F, Porto L, Weir J, Arteaga M, Padrón G, et al Epidemiology of hepatitis C virus in western Venezuela: lack of specific antibody in Indian communities. J Med Virol. 1994;43(3):287–290. . 7523583 10.1002/jmv.1890430317

[21] de Paula VS, Arruda ME, Vitral CL, Gaspar AM. Seroprevalence of viral hepatitis in riverine communities from the Western Region of the Brazilian Amazon Basin. Mem Inst Oswaldo Cruz. 2001;96(8):1123–1128. . 11784933 10.1590/s0074-02762001000800016

[22] Aguilar JI, de Souza JA, Aguiar E, Oliveira JM, de Lemos ER, Yoshida CF. Low prevalence of hepatitis B and C markers in a non-Amazonian indigenous population. Braz J Infect Dis. 2002;6(5):269–270. . 12495611 10.1590/s1413-86702002000500010

[23] Villalba JA, Bello G, Maes M, Sulbaran YF, Garzaro D, Loureiro CL, et al HIV-1 epidemic in Warao Amerindians from Venezuela: spatial phylodynamics and epidemiological patterns. AIDS. 2013;27(11):1783–1791. doi: 10.1097/QAD.0b013e3283601bdb 23435304 10.1097/QAD.0b013e3283601bdb

[24] Rangel HR, Maes M, Villalba J, Sulbarán Y, de Waard JH, Bello G, et al Evidence of at least two introductions of HIV-1 in the Amerindian Warao population from Venezuela. PLoS One. 2012;7(7):e40626 doi: 10.1371/journal.pone.0040626 22808212 10.1371/journal.pone.0040626 PMC3395626

[25] Instituto Nacional de Estadística. XIII Censo Nacional de Población y Vivienda, 2011 Caracas: Instituto Nacional de Estadística Available from: http://www.ine.gov.ve/documentos/SEN/menuSEN/pdf/subcomitedemografica/Indigena/BoletinPoblacionIndigena.pdf.

[26] Briggs CL, Mantini-Briggs C. Stories in the Time of Cholera: Racial Profiling during a Medical Nightmare. University of California Press, Los Angeles, California.Editor: Univ of California Press; 2002.

[27] Briggs CL. Poéticas de vida en espacios de muerte. Género, poder y estado en la cotidianeidad Warao. Abya–Yala, Quito Press; 2008.

[28] Ayala Lafée C, Wilbert W. La mujer Warao de recolectora deltana a recolectora urbana Fundación La Salle de Ciencias Naturales, Caracas. Press; 2008.

[29] Gutiérrez C, Devesa M, Loureiro CL, León G, Liprandi F, Pujol FH. Molecular and serological evaluation of surface antigen negative hepatitis B virus infection in blood donors from Venezuela. J Med Virol. 2004;73(2):200–7. doi: 10.1002/jmv.20076 15122793 10.1002/jmv.20076

[30] Günther S, Li BC, Miska S, Krüger DH, Meisel H, Will H. A novel method for efficient amplification of whole hepatitis B virus genomes permits rapid functional analysis and reveals deletion mutants in immunosuppressed patients. J Virol. 1995;69(9):5437–5444. . 7636989 10.1128/jvi.69.9.5437-5444.1995 PMC189390

[31] Hu X, Margolis HS, Purcell RH, Ebert J, Robertson BH. Identification of hepatitis B virus indigenous to chimpanzees. Proc Natl Acad Sci U S A. 2000;97(4):1661–1664. . 10677515 10.1073/pnas.97.4.1661 PMC26492

[32] Pujol FH, Loureiro CL. Replacement of hepatitis C virus genotype 1b by genotype 2 over a 10-year period in Venezuela. J Clin Gastroenterol. 2007;41(5):518–520. doi: 10.1097/01.mcg.0000248010.55149.ce 17450037 10.1097/01.mcg.0000248010.55149.ce

[33] Kumar S, Stecher G, Tamura K. MEGA7: Molecular Evolutionary Genetics Analysis version 7.0 for bigger dataset. Mol Biol and Evol. 2016; 33:1870–1874. 27004904 10.1093/molbev/msw054 PMC8210823

[34] Devesa M, Rodriguez C, Leon G, Liprandi F, Pujol FH. Clade analysis and surface antigen polymorphism of hepatitis B virus American genotypes. J Med Virol 2004; 72:377–384. doi: 10.1002/jmv.20015 14748061 10.1002/jmv.20015

[35] Biswas S, Candotti D, Allain JP. Specific amino acid substitutions in the S protein prevent its excretion in vitro and may contribute to occult hepatitis B virus infection. J Virol. 2013;87(14):7882–7892. doi: 10.1128/JVI.00710-13 23658444 10.1128/JVI.00710-13 PMC3700177

[36] Zhu HL, Li X, Li J, Zhang ZH. Genetic variation of occult hepatitis B virus infection. World J Gastroenterol. 2016;22(13):3531–3546. doi: 10.3748/wjg.v22.i13.3531 27053845 10.3748/wjg.v22.i13.3531 PMC4814639

[37] Carman WF, Zanetti AR, Karayiannis P, Waters J, Manzillo G, Tanzi E, et al Vaccine-induced escape mutant of hepatitis B virus. Lancet. 1990;336(8711):325–329. . 1697396 10.1016/0140-6736(90)91874-a

[38] El Chaar M, Candotti D, Crowther RA, Allain JP. Impact of hepatitis B virus surface protein mutations on the diagnosis of occult hepatitis B virus infection. Hepatology. 2010;52(5):1600–1610. doi: 10.1002/hep.23886 20815025 10.1002/hep.23886

[39] Huang CH, Yuan Q, Chen PJ, Zhang YL, Chen CR, Zheng QB, et al Influence of mutations in hepatitis B virus surface protein on viral antigenicity and phenotype in occult HBV strains from blood donors. J Hepatol. 2012;57(4):720–729. doi: 10.1016/j.jhep.2012.05.009 22634131 10.1016/j.jhep.2012.05.009

[40] Allen MI, Deslauriers M, Andrews CW, Tipples GA, Walters KA, Tyrrell DL, et al Identification and characterization of mutations in hepatitis B virus resistant to lamivudine. Lamivudine Clinical Investigation Group. Hepatology. 1998;27(6):1670–1677. doi: 10.1002/hep.510270628 9620341 10.1002/hep.510270628

[41] Flores J, Jaimes E. Prevalencia de Hepatitis viral B y Delta en población indígena del Territorio Federal Delta Amacuro. Bionotas 1989;10:34.

[42] Flores JDR, Acevedo N, Albornoz I, Martínez D. Marcadores de Hepatitis viral B y Delta en población indígena del estado Delta Amacuro. Rev Inst Nac Hig. “Rafael Rangel” 1997;28: 18–22.

[43] Allard O. Indigenous peoples and gender identities: questioning sexual dualism. Sexología y Sociedad 2013;19 (1): 64–73. ISSN 1682-0045

[44] Layrisse M, Heinen D, Salas G. Demografía de los indígenas warao. Antropológica 1977; 45–70.

[45] Torres JR, Mondolfi A. Protracted outbreak of severe delta hepatitis: experience in an isolated Amerindian population of the Upper Orinoco basin. Rev Infect Dis. 1991;13(1):52–55. . 2017631 10.1093/clinids/13.1.52

[46] Coimbra Júnior CE, Santos RV, Yoshida CF, Baptista ML, Flowers NM, do Valle AC. Hepatitis B epidemiology and cultural practices in Amerindian populations of Amazonia: the Tupí-Mondé and the Xavánte from Brazil. Soc Sci Med. 1996;42(12):1735–1743. . 8783434 10.1016/0277-9536(95)00295-2

[47] Rojo AEV. Manifestaciones religiosas de los waraos, y mitología fundante Universidad Catolica Andres 2000.

[48] Gutiérrez C, León G, Loureiro CL, Uzcátegui N, Liprandi F, Pujol FH. Hepatitis B virus DNA in blood samples positive for antibodies to core antigen and negative for surface antigen. Clin Diagn Lab Immunol. 1999;6(5):768–770. . 10473534 10.1128/cdli.6.5.768-770.1999 PMC95771

[49] Orlando R, Foggia M, Maraolo AE, Mascolo S, Palmiero G, Tambaro O, et al Prevention of hepatitis B virus infection: from the past to the future. Eur J Clin Microbiol Infect Dis. 2015;34(6):1059–1070. doi: 10.1007/s10096-015-2341-x 25678010 10.1007/s10096-015-2341-x

[50] Jaspe RC, Sulbarán YF, Loureiro CL, Martínez N, Devesa M, Rodríguez Y, et al Genetic diversity of hepatitis B virus and hepatitis C virus in human immunodeficiency virus type 1-co-infected patients from Venezuela. J Med Microbiol. 2014;63(Pt 8):1099–1104. doi: 10.1099/jmm.0.067496-0 24895404 10.1099/jmm.0.067496-0

[51] Bautista-Amorocho H, Castellanos-Domínguez YZ, Rodríguez-Villamizar LA, Velandia-Cruz SA, Becerra-Peña JA, Farfán-García AE. Epidemiology, risk factors and genotypes of HBV in HIV-infected patients in the Northeast region of Colombia: High prevalence of occult hepatitis B and F3 subgenotype dominance. PLoS One 2014;9(12):e114272 doi: 10.1371/journal.pone.0114272 eCollection 2014. 25462190 10.1371/journal.pone.0114272 PMC4252145

[52] Maes M, Kremer K, van Soolingen D, Takiff H, de Waard JH. 24-locus MIRU-VNTR genotyping is a useful tool to study the molecular epidemiology of tuberculosis among Warao Amerindians in Venezuela. Tuberculosis (Edinb). 2008;88(5):490–494. doi: 10.1016/j.tube.2008.04.003 18514577 10.1016/j.tube.2008.04.003

[53] Verhagen LM, Incani RN, Franco CR, Ugarte A, Cadenas Y, Sierra Ruiz CI, et al High malnutrition rate in Venezuelan Yanomami compared to Warao Amerindians and Creoles: significant associations with intestinal parasites and anemia. PLoS One. 2013;8(10):e77581 doi: 10.1371/journal.pone.0077581 24143243 10.1371/journal.pone.0077581 PMC3797096

[54] Trigo C, do Brasil PE, Costa MJ, de Castro L. Occult hepatitis B virus infection: clinical implications in tuberculosis treatment. J Viral Hepat. 2016;23(12):1027–1035. doi: 10.1111/jvh.12583 27624908 10.1111/jvh.12583

[55] Delfino CM, Eirin ME, Berini C, Malan R, Gentile E, Castillo A, et al HDAg-L variants in covert hepatitis D and HBV occult infection among Amerindians of Argentina: new insights. J Clin Virol. 2012;54(3):223–228. doi: 10.1016/j.jcv.2012.04.014 22608280 10.1016/j.jcv.2012.04.014

[56] Navas MC, Jaramillo CM, de la Hoz F, Cortes-Mancera F, Choconta LA, Payares E, et al Identification of escape mutants in isolates of Hepatitis B virus (HBV) from Amerindian communities in Amazonas, Colombia. America Society for Virology Meeting. 2013; P31–11.

[57] Ariza H, Gutiérrez C, Amelis G. Infección residual del virus de la hepatitis B en pacientes infectados por el virus de inmunodeficiencia humana en Venezuela. Rev Panam Infectol. 2009; 11(1): 27–32.

[58] Puche ML, Kay-Valero S, Michelli P, Oropeza MD, Loureiro CL, Devesa M, et al Genetic diversity of hepatitis B virus and mutations associated to hepatocellular carcinoma in patients from Venezuela, with different stages of liver disease. Invest Clin. 2016;57(1):38–46. . 27382800

